# Vitamin D Deficiency as a Risk Factor for Diabetes and Poor Glycemic Control in Saudi Arabia: A Systematic Review

**DOI:** 10.7759/cureus.48577

**Published:** 2023-11-09

**Authors:** Abdelrahman Mohamed A Abukanna, Reem Falah A Alanazi, Fatimah S Alruwaili, Asma Zaid M Alayyashi, Farah Alanzi

**Affiliations:** 1 Internal Medicine, Faculty of Medicine, Northern Border University (NBU), Arar, SAU; 2 Medicine, Faculty of Medicine, Northern Border University (NBU), Arar, SAU

**Keywords:** poor glycemic control, saudi arabia systematic review, t2dm, diabetes mellitus, vitamin d deficiency

## Abstract

This systematic review aims to investigate the published literature on vitamin D deficiency (VDD) as a risk factor for developing type 2 diabetes mellitus (T2DM) or affecting the glycemic state of patients in Saudi Arabia. PubMed, Scopus, Web of Science, ScienceDirect, and the Cochrane Library were systematically searched to include the relevant literature. Rayyan QCRI (Rayyan Systems Inc., Cambridge, MA) was used throughout this systematic approach. Eleven studies were included with a total of 4229 patients. Three studies demonstrated that VDD was a significant risk factor for developing T2DM, and one reported that it increased insulin resistance. However, two studies found that VDD did not affect the incidence of T2DM and did not affect the insulin sensitivity or glycated hemoglobin (HbA1c) levels in patients with T2DM. This systematic review demonstrated that VDD significantly increases the risk of developing T2DM and negatively affects the glycemic state of patients with T2DM among Saudi patients. Due to the many populations examined, vitamin D chemical compositions, doses, and supplementation periods, interventional research has produced contradictory and ambiguous results. Additional research is necessary, particularly in individuals with a high risk of developing diabetes (impaired fasting glucose and/or glucose tolerance, possibly without obesity). These individuals may be the primary benefactors of vitamin D's benefits in preventing T2DM, according to the hypothesized mechanism of action for the vitamin.

## Introduction and background

The prevalence of type 2 diabetes mellitus (T2DM), a chronic metabolic disorder, has considerably grown in emerging nations [1]. By 2030, there will likely be 366 million people worldwide with T2DM, more than doubling the current population [2]. Untreated T2DM can cause both macrovascular and microvascular problems, as well as other comorbidities [3]. T2DM etiology is uncertain since multiple dysfunctional pathways occur concurrently and advance the disease [4].

T2DM patients frequently have low 25-hydroxyvitamin D (25OHD) levels [5,6]. Low 25OHD levels are linked to higher fasting glucose and glycated hemoglobin (HbA1c) levels in people with established diabetes mellitus and in the general population [7]. Low 25OHD levels are also linked to an increased likelihood of developing diabetes mellitus or metabolic syndrome in the future, according to prospective population studies [8,9].

There are a number of routes by which vitamin D could impact glucose metabolism. The anti-inflammatory and immunomodulatory properties of vitamin D have been documented [10]. This might affect type 1 diabetes' autoimmunity pathology and T2DM's low-grade chronic inflammation, which has been linked to insulin resistance [11]. Additionally, pancreatic cells may secrete insulin when vitamin D is present [12,13]. Low vitamin D levels have been linked to elevated parathyroid hormone (PTH) levels, which have been linked to decreased insulin release from pancreatic beta-cells [14].

A number of studies have discovered a link between vitamin D deficiency (VDD) and the "metabolic syndrome," a collection of metabolic abnormalities that include abdominal obesity, insulin resistance, dyslipidemia, and hypertension, as well as a higher risk of developing cardiovascular disease and/or type 2 diabetes (T2D) [15]. Intriguingly, several investigations have revealed that vitamin D may play a role in the inhibition of cell secretion. The idea of the potential involvement of vitamin D in the etiology of T2DM was created as a result of the simultaneous link between VDD and insulin resistance, decreased insulin production, and their significant metabolic repercussions [16,17]. This systematic review aims to investigate the published literature on VDD as a risk factor for developing T2DM or affecting the glycemic state of patients in Saudi Arabia.

## Review

This systematic review was conducted in accordance with accepted standards (Preferred Reporting Items for Systematic Reviews and Meta-Analyses, PRISMA) [18]. This was conducted between August and September 2023. A thorough search of five major databases, i.e., PubMed, Scopus, Web of Science, ScienceDirect, and the Cochrane Library, was done to find the relevant literature. We restricted our search to English and considered each database's unique requirements. The following keywords were converted into PubMed Medical Subject Heading (MeSH) terms and used to find the relevant studies: "Vitamin D," "25 (OH) D," "Diabetes," "T2DM Prediabetes," "Glycemic status," and "Risk." The Boolean operators "OR" and "AND" matched the required keywords. Publications with full English text, available free articles, and human trials were among the search results.

We considered the following criteria for inclusion in this review: study designs that investigated the published literature on VDD as a risk factor for developing T2DM or affecting the glycemic state of patients in Saudi Arabia; studies that included the estimation of the risk of developing T2DM or worsening the glycemic status, not only assessing the prevalence of VDD among diabetic patients; T2DM patients; and free accessible articles. The exclusion criteria were type 1 diabetes mellitus (T1DM) and gestational diabetes, children (<18 years), and case reports.

The search strategy's output was checked for duplication using Rayyan QCRI (Rayyan Systems Inc., Cambridge, MA) [19]. The researchers evaluated the relevance of the titles and abstracts by modifying the combined search results using a set of inclusion/exclusion criteria. The reviewers carefully examined each paper that met the criteria for inclusion. Techniques for resolving disputes were covered by the authors. With the use of a previously created data extraction form, the authorized study was uploaded. The authors extracted data about the study titles, authors, study year, city or region, participants, gender, objectives, and main outcomes. A separate sheet was created for the risk of bias assessment.

To give a qualitative analysis of the findings and study components, summary tables were made utilizing data from relevant research. Once the data for the systematic review were retrieved, the most efficient way to use the data from the included study articles was chosen.

The Risk of Bias in Non-randomized Studies of Interventions (ROBINS-I) risk of bias assessment method for non-randomized trials of treatments was used to assess the quality of the included studies [20]. The seven topics that were assessed included confounding, participant selection for the study, the classification of interventions, deviations from intended interventions, missing data, the assessment of outcomes, and the selection of the reported result.

Results

**Figure 1:**
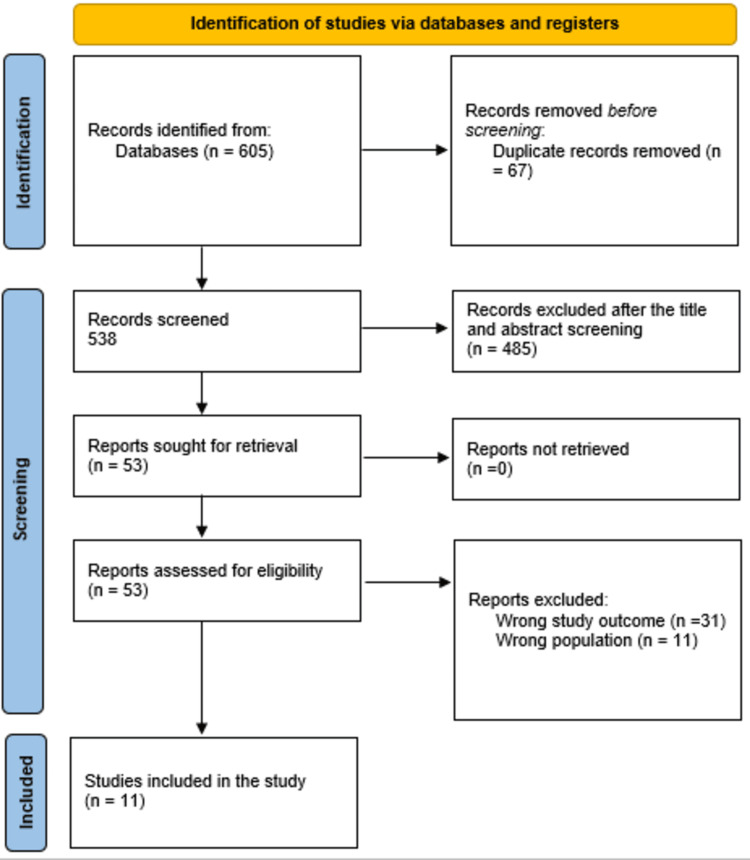
PRISMA flowchart summarizing the study selection process. PRISMA: Preferred Reporting Items for Systematic Reviews and Meta-Analyses

A total of 605 study articles resulted from the systematic search, and 67 duplicates were deleted. Title and abstract screening was conducted on 538 studies, and 485 studies were excluded. Fifty-three reports were sought for retrieval, and none were retrieved. Finally, 53 studies were screened for full-text assessment; 31 were excluded for wrong study outcomes and 11 for the wrong population type. Eleven study articles were included in this systematic review. A summary of the study selection process is presented in Figure 1.

Characteristics of the included studies

Table 1 presents the sociodemographic characteristics of the included study articles. Our results included 11 studies with a total of 4229 subjects [21-31]. Two studies were conducted in Arar [30,31], one in the Eastern Region [21], one in Taif [22], one in Jeddah [23], one being multi-centered [24], one in Jazan [25], one in Tabuk [26], one in Al Kharj [27], one in the Southern Region [28], and one in Riyadh [29]. Five studies were cross-sectional [23-26,30], four were case-control studies [21,22,28,31], one was retrospective in nature [27], and one was a randomized controlled trial (RCT) [29].

**Table 1:** Sociodemographic characteristics of the included participants. NM, not mentioned; RCT, randomized controlled trial

Study	Study design	Location	Participants	Mean age (years)	Males (%)
Giha et al., 2022 [21]	Case-control	Eastern Region	104	48.3 ± 10.8	59 (56.7)
Alkhedaide et al., 2021 [22]	Case-control	Taif	145	NM	NM
Alharazy et al., 2021 [23]	Cross-sectional	Jeddah	173	59.6 ± 6.8	0
Al-Sumaih et al., 2020 [24]	Cross-sectional	Multi-centered	2084	39.22 ± 16.43	1127 (54.1)
Darraj et al., 2019 [25]	Cross-sectional	Jazan	309	58.9 ± 12	130 (42.1)
Alloubani et al., 2019 [26]	Cross-sectional	Tabuk	350	18-60 (range)	150 (42.9)
Al Dossari et al., 2020 [27]	Retrospective	Al Kharj	200	42.4 ± 14.8	118 (59)
Alhumaidi et al., 2013 [28]	Case-control	Southern Region	345	53.4 + 15.6	171 (49.6)
Al-Sofiani et al., 2015 [29]	RCT	Riyadh	20	54.8 ± 9.16	15 (75)
el-Fetoh et al., 2017 [30]	Cross-sectional	Arar	439	27 ± 4.3	125 (71.5)
Alruwaili, 2018 [31]	Case-control	Arar	60	45 (median)	10 (16.7)

Table 2 presents the clinical characteristics. Three studies demonstrated that VDD was a significant risk factor for developing T2DM [27,30,31], and one study reported that it increased insulin resistance [23]. Other reported studies found that patients with T2DM, as well as VDD, had poor glycemic control statuses [21,25]. However, two studies found that VDD did not affect the incidence of T2DM [24,28] and did not affect the insulin sensitivity or HbA1c levels in patients with T2DM [29].

**Table 2:** Clinical characteristics and outcomes of the included studies. NA, not applicable; T2DM, type 2 diabetes mellitus; ROBIN-I, Risk of Bias in Non-randomized Studies of Interventions; VDD, vitamin D deficiency; DM, diabetes mellitus; 25OH, 25-hydroxy; HbA1c, glycated hemoglobin

Study	Objectives	Type of patients	Main outcomes	ROBIN-I
Giha et al., 2022 [21]	To study the hormonal and metabolic profiles of obese and non-obese T2DM patients	Obese and non-obese patients with T2DM	Female sex, young people, short illness duration, significant deficiencies in vitamin D, and a predisposition toward high insulin levels were all significantly linked to diabetic obesity	Moderate
Alkhedaide et al., 2021 [22]	To explore any potential links between vitamin D receptor genetic polymorphism at the Apa1 and Taq1 loci and the development of T2DM	The general population with T2DM	According to the most recent data, T2DM is statistically related to the T allele of Apa1 (rs7975232) and the T allele of Taq1 (rs731236) of the vitamin D receptor, whereas the G allele of Apa1 (rs7975232) and the C allele of Taq1 (rs731236) of the vitamin D receptor are protecting alleles	Moderate
Alharazy et al., 2021 [23]	To determine whether ethnicity has an impact on the relationship between vitamin D and glycemic profile, as well as markers of obesity as a secondary outcome	Postmenopausal females with T2DM	Vitamin D status in T2DM was correlated with insulin resistance and obesity. Females from White racial backgrounds have different vitamin D associations than those from Black or Asian ones. It is important to look into whether vitamin D supplementation can reduce obesity or increase insulin sensitivity in various ethnic populations	High
Al-Sumaih et al., 2020 [24]	To investigate the connections between T2DM, hypertension, vitamin D, and obesity in Saudi nationals aged 15 and older	The general population with T2DM	No correlation between vitamin D levels and the incidence of diabetes or hypertension was discovered	Moderate
Darraj et al., 2019 [25]	To estimate the prevalence of VDD in Jazan T2DM patients and research its relationships to patient traits and glycemic management	The general population with T2DM	VDD is quite common in T2DM patients and is linked to inadequate glycemic management. Healthcare professionals may be able to provide T2DM patients with better outcomes by diagnosing VDD earlier	Moderate
Alloubani et al., 2019 [26]	To determine the lifestyle and dietary habits of Saudi Arabians in order to determine the prevalence of VDD and the relationship between VDD, DM, and obesity	The general population with T2DM	In Saudi people of various ages, VDD was common in both males and females. It is necessary to emphasize the importance of recognizing VDD screening for risk factors due to the link between VDD and major chronic diseases (including T2DM)	High
Al Dossari et al., 2020 [27]	To determine whether the vitamin D level in Saudi patients with T2DM affects glycemic management in any way	The general population with T2DM	The development of T2DM may be significantly influenced by the low vitamin D level. Most people do not get enough 25OH vitamin D. They discovered a link between low vitamin D levels and poor glycemic control in T2DM patients	Moderate
Alhumaidi et al., 2013 [28]	To calculate the level of 25OH VDD in T2DM patients in contrast to the healthy, age-matched nondiabetic control population	The general population with T2DM	The overall conclusion that 98.5% of the population lacks 25OH vitamin D cannot be disregarded. They could not find any significant difference in vitamin D status between patients with and without diabetes	NA
Al-Sofiani et al., 2015 [29]	To investigate whether vitamin D supplementation could enhance glucose metabolism, elements of the metabolic syndrome (MetS), and specific inflammatory biomarkers in people with T2DM and VDD	The general population with T2DM	With no discernible changes in HbA1c or insulin sensitivity, vitamin D repletion for 12 weeks improved serum vitamin D concentrations and enhanced cell activity in vitamin D-deficient T2DM	Moderate
el-Fetoh et al., 2017 [30]	To demonstrate VDD and how it may lead to T2DM, as well as obesity and overweight	The general population with T2DM	DM is strongly linked to VDD. T2DM is more common in cases of VDD, affecting 16.3% of those cases compared to 10.3% of participants with normal vitamin D levels. This difference is statistically significant (P = 0.05)	Moderate
Alruwaili, 2018 [31]	To investigate whether low vitamin D levels and prediabetes might be related	The general population with T2DM	Prediabetics should take vitamin D supplements since those who are vitamin D-deficient have a higher chance of acquiring T2DM	-

Discussion

Recent strong evidence points to a role for VDD in the etiology of insulin resistance and insulin secretion abnormalities, with potential interference with T2DM as a result. Uncertainty surrounds the mechanism of this relationship [32,33]. This systematic review aims to investigate the published literature on VDD as a risk factor for developing T2DM or affecting the glycemic state of patients in Saudi Arabia.

We found that VDD was a significant risk factor for developing T2DM [27,30,31], and one study reported that it increased insulin resistance [23]. Other reported studies found that patients with T2Dm and VDD had poor glycemic control statuses [21,25]. As Albannawi et al. [33] conducted a similar systematic review and reported a high prevalence of VDD among T2DM patients in Saudi Arabia, the mechanism of these associations needs further understanding.

Based on research indicating the existence of vitamin D receptors in tissues other than bone, gut, and kidneys, there has recently been an increase in interest in the nonclassical effects of vitamin D [34]. Additionally, a number of studies have revealed that vitamin D may play a role in the development of cancer, metabolic syndrome, and cardiovascular illnesses [35-37]. There is a significantly increased risk of T2DM and impaired glucose metabolism in settings of VDD, according to numerous cross-sectional studies, though not consistently [38].

According to the excursus of several research mentioned above, VDD is significantly linked to obesity, mostly as a result of the 25OHD vitamin's lipophilic characteristics, which allow it to be stored in adipose tissue. A decrease in calcium concentration, a rise in PTH, and a direct influence of vitamin D on deteriorating insulin resistance and secretion, which increases the likelihood of developing T2DM, are just a few of the mechanisms that could cause a drop in 25OHD levels. Due to the many populations examined, vitamin D chemical compositions, doses, and supplementation periods, interventional research has produced contradictory and ambiguous results [32].

There is currently insufficient evidence to support the recommendation of vitamin D supplementation as a foundation to improve insulin resistance and glycemia in patients with diabetes, normal fasting glucose, and impaired glucose tolerance, according to a meta-analysis and systematic review of 15 studies looking at the effects of vitamin D supplementation [39]. The effects of vitamin D supplementation were investigated in a meta-analysis/systematic review of 15 research studies. The findings indicated insufficient support for vitamin D supplementation's usefulness in treating patients with normal fasting glucose, impaired glucose tolerance, or diabetes [39].

## Conclusions

This systematic review demonstrated that VDD significantly increases the risk of developing T2DM and negatively affects the glycemic state of patients with T2DM among Saudi patients. Due to the many populations examined, vitamin D chemical compositions, doses, and supplementation periods, interventional research has produced contradictory and ambiguous results. Additional research is necessary, particularly in individuals who have a high risk of developing diabetes (impaired fasting glucose and/or glucose tolerance, possibly without obesity). These individuals may be the primary benefactors of vitamin D's benefits in preventing T2DM, according to the hypothesized mechanism of action for the vitamin.
